# Environmental filtering drives distinct continental atlases of soil archaea between dryland and wetland agricultural ecosystems

**DOI:** 10.1186/s40168-019-0630-9

**Published:** 2019-02-01

**Authors:** Shuo Jiao, Yiqin Xu, Jie Zhang, Yahai Lu

**Affiliations:** College of Urban and Environmental Sciences, Peking University, Beijing, 100871 People’s Republic of China

**Keywords:** Archaea, Continental atlas, Agricultural ecosystem, Environmental filtering

## Abstract

**Background:**

Understanding the spatial distributions and ecological diversity of soil archaeal communities in agricultural ecosystems is crucial for improvements in crop productivity. Here, we conducted a comprehensive, continental-scale survey of soil archaeal communities in adjacent pairs of maize (dryland) and rice (wetland) fields in eastern China.

**Results:**

We revealed the consequential roles of environmental filtering in driving archaeal community assembly for both maize and rice fields. Rice fields, abundant with Euryarchaeota, had higher archaeal diversity and steeper distance-decay slopes than maize fields dominated by Thaumarchaeota. Dominant soil archaea showed distinct continental atlases and niche differentiation between dryland and wetland habitats, where they were associated with soil pH and mean annual temperature, respectively. After identifying their environmental preferences, we grouped the dominant archaeal taxa into different ecological clusters and determined the unique co-occurrence patterns within each cluster. Using this empirical dataset, we built a continental atlas of soil archaeal communities to provide reliable estimates of their spatial distributions in agricultural ecosystems.

**Conclusions:**

Environmental filtering plays a crucial role in driving the distinct continental atlases of dominant soil archaeal communities between dryland and wetland, with contrasting strategies of archaeal-driven nutrient cycling within these two agricultural ecosystems. These findings improve our ability to predict how soil archaeal communities respond to environmental changes and to manage soil archaeal communities for provisioning of agricultural ecosystem services.

**Electronic supplementary material:**

The online version of this article (10.1186/s40168-019-0630-9) contains supplementary material, which is available to authorized users.

## Background

Soil microorganisms represent one of the largest reservoirs of biodiversity and drive a variety of ecological processes in terrestrial ecosystems [1–3]. In particular, archaea, the third domain of life, constitute a considerable fraction of the soil microbial community and its biomass [4–6]. Euryarchaeota and Thaumarchaeota (formerly described as mesophilic Crenarchaeota [7]) are two major archaeal phyla, and both were recently found to contribute to the biogeochemical cycling of carbon, nitrogen, and hydrogen [8]; for example, euryarchaeotal methanogenesis is typically considered the dominant process in anaerobic habitats [9]. Moreover, the autotrophic ammonia-oxidizing archaea, which belong to Thaumarchaeota, possess homologs of the alpha and beta subunits of the bacterial ammonia monooxygenase enzyme and predominate among ammonia-oxidizing prokaryotes in soils [10, 11]. Ammonia oxidation is the first step in nitrification, a key process in the global nitrogen cycle that results in the formation of nitrate through microbial activity [10].

Studies to date of terrestrial archaeal ecology have focused mainly on natural ecosystems, finding high variability in the general structure and relative abundance of soil archaeal communities along broad environmental gradients and among various habitat types [4–6]. Agricultural fields are typical human-managed terrestrial ecosystems and are vital for securing a global food supply. Importantly, agricultural ecosystems can make substantial contributions to water and soil conservation, climate regulation, and atmospheric quality [12]. Due to long-term tillage and fertilization practices, soil physicochemical properties and ecosystem processes tend to diverge between agricultural and natural ecosystems, resulting in distinct microbial assembly patterns [13]. Furthermore, agricultural ecosystems are influenced by varying water management practices, such as occasional irrigation in maize fields (dryland) and continuous flooding in rice paddies (wetland). Given their pronounced difference in oxygenation levels, microbially mediated soil processes and ecological diversity patterns are also likely to be distinct between wetland and dryland soils. In addition, the emissions of greenhouse gases (e.g., methane and nitrous oxide) during rice cultivation are of great concern [14]. Since soil archaea play important roles in ecosystem biogeochemical cycles, they are crucial for nutrient management, crop productivity, and climate regulation [15]. Therefore, it is necessary to reveal the fundamental processes that underlie archaeal biogeographic and ecological diversity patterns in these two widely used yet distinctive agricultural ecosystems.

Uncovering the spatial assembly of microbial communities remains a major challenge in microbial ecology [16]. The focus of microbial biogeography is now turning towards the underlying processes in microbial assembly from an initial focus on the description of patterns [17, 18]. The recent literature has instilled a growing awareness that archaeal communities are affected by various soil and environmental factors, including soil pH [6, 19], climate [20], nutrient availability [5], and spatial distance [21]. However, because the available studies are confined to specific sites, it is difficult to obtain a systematic and detailed ecological understanding of archaeal community distributions in soils across large-scale regions. Unraveling the ecological attributes of particular dominant archaeal taxa could help us predict how soil archaeal communities shift spatially and temporally, as well as how agricultural ecosystems respond to current and future environmental changes. Recently, a global atlas of dominant soil bacteria was compiled, and a “most wanted” list of taxa was narrowed down to illustrate the spatial distribution of soil bacteria and their contribution to ecosystem functioning [22]. Since microbial life-history depends on oxygen conditions—archaea are mainly anaerobic whereas bacteria are mainly aerobic [9]—the spatial distributions of soil archaea and bacteria may as well be very different. Yet, we currently lack a predictive ecological understanding of soil individual archaeal taxa and know little about their environmental preferences, traits, and metabolic capabilities. An outstanding issue is whether dominant archaeal communities can exist abundantly and ubiquitously in agricultural soils across distant sites. Resolving this critical issue would advance our understanding of soil archaeal communities.

Maize and rice are two major crops widely cultivated across China, thus making them excellent models for assessing broad-scale questions about microbial diversity and assembly between dryland and wetland agricultural ecosystems. Here, we carried out a large-scale soil survey of archaeal communities in agricultural soils from adjacent pairs of maize and rice cultivated fields across eastern China, aimed at controlling for the influences of spatial scale and climatic factors on patterns of archaeal diversity. The objectives of the present study were (1) to explore the relative contributions of underlying factors to archaeal assembly, (2) to identify the dominant archaeal taxa and determine their habitat preferences and co-occurrence patterns, and (3) to construct a continental atlas of archaeal spatial distributions in wetland and dryland agricultural ecosystems. Our study suggests that the spatial distributions of the dominant archaeal taxa in agricultural ecosystems are predictable, which should improve our ability to manage soil archaeal communities to provide key ecosystem services and improve crop productivity.

## Results

From all the 249 samples, we obtained a total of 18,105,503 high-quality sequences, which clustered into 3509 operational taxonomic units (OTUs). The majority of these sequences belonged to the phyla Thaumarchaeota (54.9%) and Euryarchaeota (40.0%), while Bathyarchaeota, Parvarchaeota, Lokiarchaeota, and Hadesarchaea were detected at low relative abundances.

The archaeal *α*-diversity indices were significantly higher for soils from rice fields than those from maize fields (*p* < 0.001; Additional file 1: Figure S2). The edaphic and climatic drivers of this diversity were not the same between the fields, however (multiple regression analysis, Additional file 1: Table S1). While mean annual temperatures (MAT) and carbon-nitrogen ratios (C/N ratios) contributed the most towards explaining the variation in the Shannon index of maize fields, available iron (AFe) and MAT contributed most for rice fields. This result was confirmed by significant and negative simple linear regressions found between MAT and Shannon index for both maize and rice field samples, whereas the Shannon index of soil samples from maize and rice fields increased respectively with greater C/N ratios and AFe (Additional file 1: Figure S3).

**Fig. 1:**
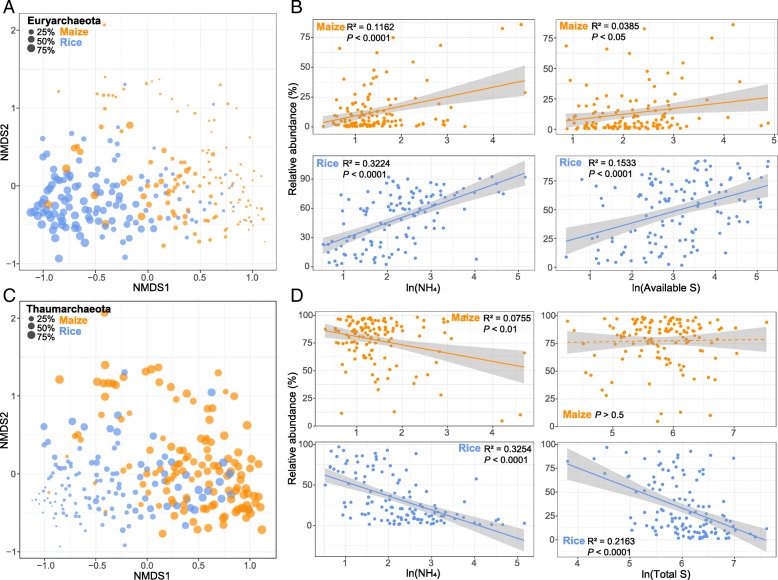
General distributions of major archaeal phyla in soil samples from maize and rice fields across eastern China. Nonmetric multidimensional scaling (NMDS) showed the structure of archaeal community among the samples between maize and rice soils. The size of each point is proportional to the relative abundance of Euryachaeota (**a**) and Thaumarchaeota (**c**), respectively. Drivers of the distributions of Euryachaeota (**b**) and Thaumarchaeota (**d**) were estimated via linear least-squares regression analysis, including ammoniacal nitrogen (NH_4_), available sulfur (S), and total S. The contents of these environmental factors were log transformed

Soil archaeal community relationships were visualized via nonmetric multidimensional scaling (NMDS) analysis (Fig. 1a and c). These ordination graphs revealed that soil samples from maize and rice fields formed distinct clusters as confirmed by permutational multivariate analysis of variance (ADONIS; *R*^2^ = 0.1033, *p* < 0.001) and similarity analysis (ANOSIM; *R* = 0.3536, *p* < 0.001). Euryarchaeota was more abundant in rice soils, while Thaumarchaeota was mainly dominant in the maize soil samples, leading to significantly different distributions of these two phyla in relative abundance between maize and rice fields (*p* < 0.001, Wilcoxon rank-sum test; Additional file 1: Figure S4).

We also examined the influence of environmental variables on the spatial distributions of these two major phyla (Additional file 1: Table S2). In maize fields, ammonium-nitrogen (NH_4_) was the most important contributor to both phyla, yet in rice fields, it was NH_4_ and available sulfur (AS) that explained the most variation in Euryarchaeota’s relative abundance, while total sulfur (TS) and AS contributed most for Thaumarchaeota. Specifically, according to follow-up bivariate regressions, irrespective of the maize or rice, the relative abundance of Euryarchaeota increased significantly with greater NH_4_ and AS levels in soil while that of Thaumarchaeota rose with NH_4_. Nonetheless, in rice fields only, significant and negative linear regressions were found between the relative abundance of Thaumarchaeota and TS levels (Fig. 1b and d). Moreover, when we modeled the spatial distributions of these two phyla (using a kriging interpolation method), their predicted patterns showed that Euryarchaeota had a greater relative abundance in southern field than the northern counterpart, whereas this trend was reversed in Thaumarchaeota (Additional file 1: Figure S5). These results were observed in both maize and rice soils.

The relative abundances of archaeal orders and genera were estimated for the maize and rice soils (Additional file 1: Figures S6 and S7). *Methanosarcinales*, *Methanocellales*, *Methanomicrobiales*, and *Methanobacteriales* were dominant in rice soils, while the relative abundances of *unidentified_SCG* and *unidentified_SAGMCG-1* were significantly higher in maize soils. In addition, *Nitrososphaera* and *Nitrosotalea* were the dominant soil archaeal genera in maize fields, while *Methanocella*, *Methanobacterium*, *Methanosarcina*, *Methanoregula*, and *Methanosaeta* dominated the rice fields.

**Fig. 2:**
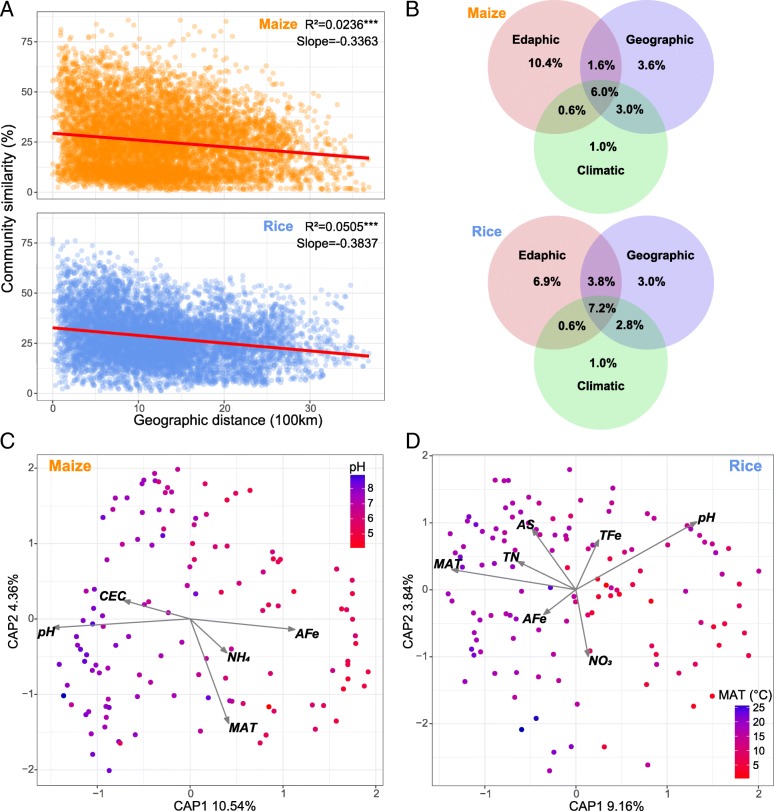
Drivers of archaeal *β*-diversity in maize and rice soil samples. **a** Distance–decay curves of similarity for soil archaeal communities. Red lines denote the ordinary least squares linear regression. Asterisks represent significance of correlation (***, *p* < 0.0001). **b** Variation partitioning analysis of the relative contributions of edaphic, geographic, and climatic variables to variation in soil archaeal *β*-diversity. **c** and **d** Constrained analysis of principal coordinates (CAP) showing edaphic and climatic factors that influenced archaeal assembly. Sample points are colored according to soil pH (left panel) and mean annual temperature (MAT; right panel). The color bar from red to blue represents values from small to large. CEC, cation exchange capacity; NH_4_, ammonium-nitrogen; NO_3_, nitrate-nitrogen; TN, total nitrogen; AFe, available iron; TFe, total iron; and AS, available sulfur

To disentangle the drivers of archaeal *β*-diversity, we first estimated the distance decay of archaeal community similarity. Significant distance-decay relationships were observed for both maize and rice soils (Fig. 2a), with the former having a steeper slope (slope = − 0.3837, *p* < 0.001) than the latter (slope = − 0.3363, *p* < 0.001). Next, we explored the main environmental variables that shaped the sampled archaeal communities. Constrained analysis of principal coordinates suggested that soil pH and MAT were the most important variables for archaeal assembly in maize and rice soils, respectively (Fig. 2c and d, Additional file 1: Tables S3 and S4). Nevertheless, several other variables, including cation exchange capacity (CEC), NH_4_, and AFe, also significantly influenced the archaeal community of maize soils while AS, nitrate-nitrogen (NO3), and total iron (TFe) were more influential in rice soils.

The contribution of edaphic, geographic, and climatic variables to archaeal community variation is illustrated with a modified variation partitioning diagram (Fig. 2b). All the variation partitioning fractions were significant in an ANOVA permutation test (*p* < 0.01), and the complete set of all variables together explained 26.2% and 25.3% of the variation in the archaeal communities of maize and rice soils, respectively, with edaphic properties clearly contributing most. Geographic factors contributed a larger proportion of variation relative to edaphic factors to the archaeal *β*-diversity of rice soils (43.5%) than that of maize soils (34.6%).

Those OTUs in the top 20% of relative abundance (i.e., highly abundant) and occurring in more than half of all soil samples (i.e., ubiquitous) were deemed dominant archaeal taxa. This amounted to 339 OTUs, accounting for 9.7% of all observed taxa. Nonetheless, on average, these dominant taxa accounted for 93.1% of the sequences. Given the distinct archaeal assembly patterns between maize and rice fields, we determined the ecological preferences of dominant archaeal taxa by focusing on whether they preferred a waterlogged environment. The dominant archaeal taxa were grouped into two ecological clusters sharing habitat preferences for (1) dryland and (2) paddy (Additional file 1: Table S5).

**Fig. 3:**
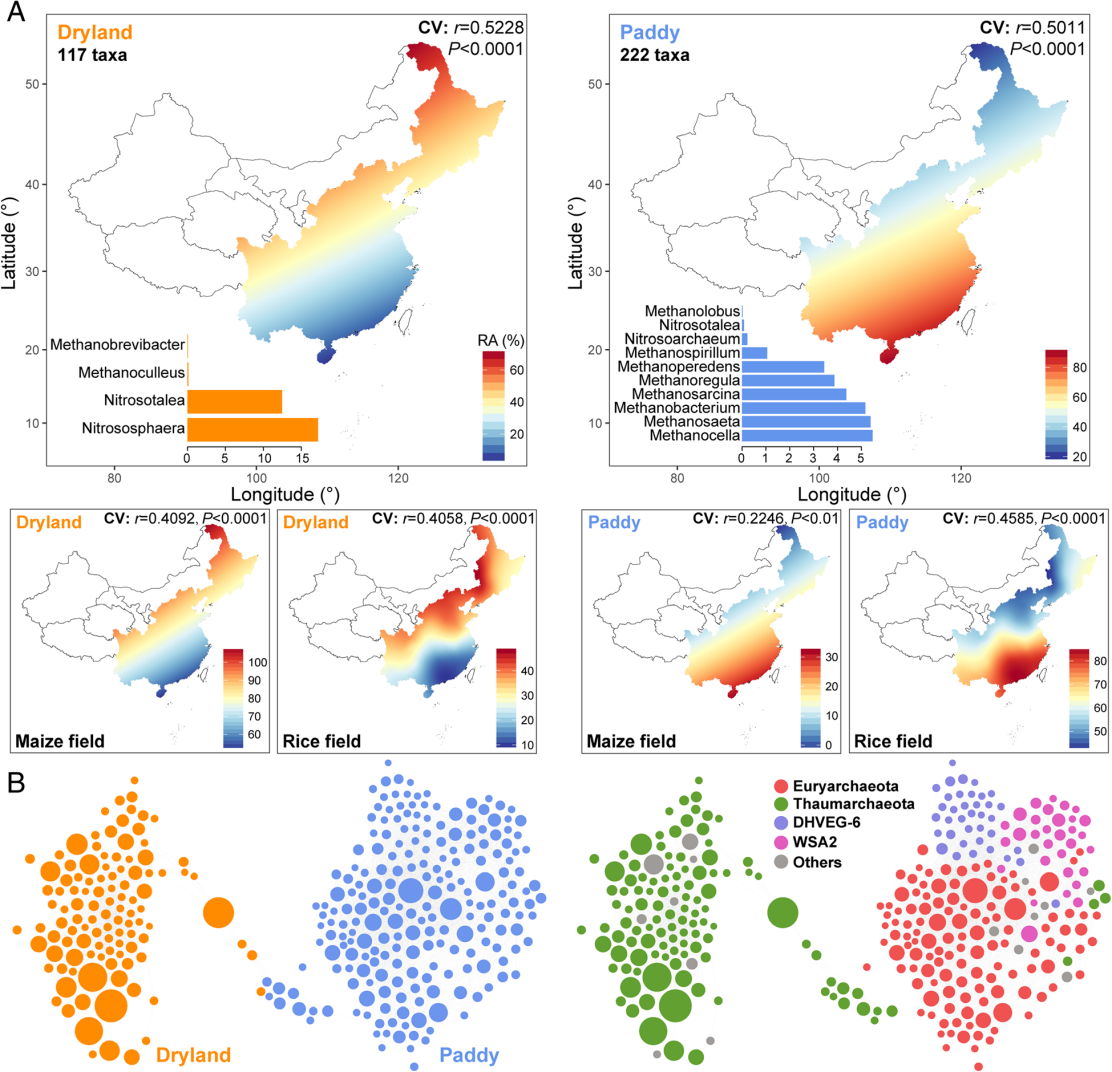
Continental atlases and co-occurrence patterns of dominant soil archaea with shared habitat preferences in agricultural fields across eastern China. Spearman correlations identified two ecological clusters of dominant taxa with shared habitat preferences, (1) dryland and (2) paddy (**a**). The above two maps predicted the distributions of dominant archaeal taxa for each identified habitat preference across all sampling fields. Taxonomic compositions at genera level for the two ecological clusters of dominant archaeal taxa were displayed at the left-bottom of each graph. The following four maps show predicted distributions of dominant archaeal taxa for each identified habitat preference in maize and rice fields, respectively. The cross-validation (“CV”) of the maps based on Pearson correlation between the predicted and observed values in each sampling site. Network diagrams with nodes (dominant archaeal taxa) colored by ecological clusters and phylum (**b**). The size of each node is proportional to the relative abundance of the taxa; the thickness of each connection between two nodes (edge) is proportional to the value of Spearman correlation coefficient

We constructed spatial distribution maps of these two ecological clusters of dominant archaeal taxa, finding that the dryland cluster had a greater relative abundance in northern than southern counterparts for either maize or rice fields, whereas this trend was reversed in the paddy cluster (Fig. 3a). Each of the ecological clusters identified included taxa belonging to multiple genera. Thaumarchaeotal *Nitrososphaera* and *Nitrosotalea* were abundant in dryland clusters. By contrast, euryarchaeotal *Methanocella*, *Methanosaeta*, *Methanobacterium*, *Methanosarcina*, and *Methanoregula* preferred paddy environments. Moreover, correlation network analyses were used to cross-validate whether archaeal taxa sharing similar habitat preferences tended to co-occur. Nodes within the same ecological clusters were more connected and generated independent modules; that is, dominant archaeal taxa sharing a particular habitat preference tend to co-occur with each other (Fig. 3b). In addition, dominant archaeal taxa within the same phyla tended to co-occur with each other (Fig. 3b).

**Fig. 4:**
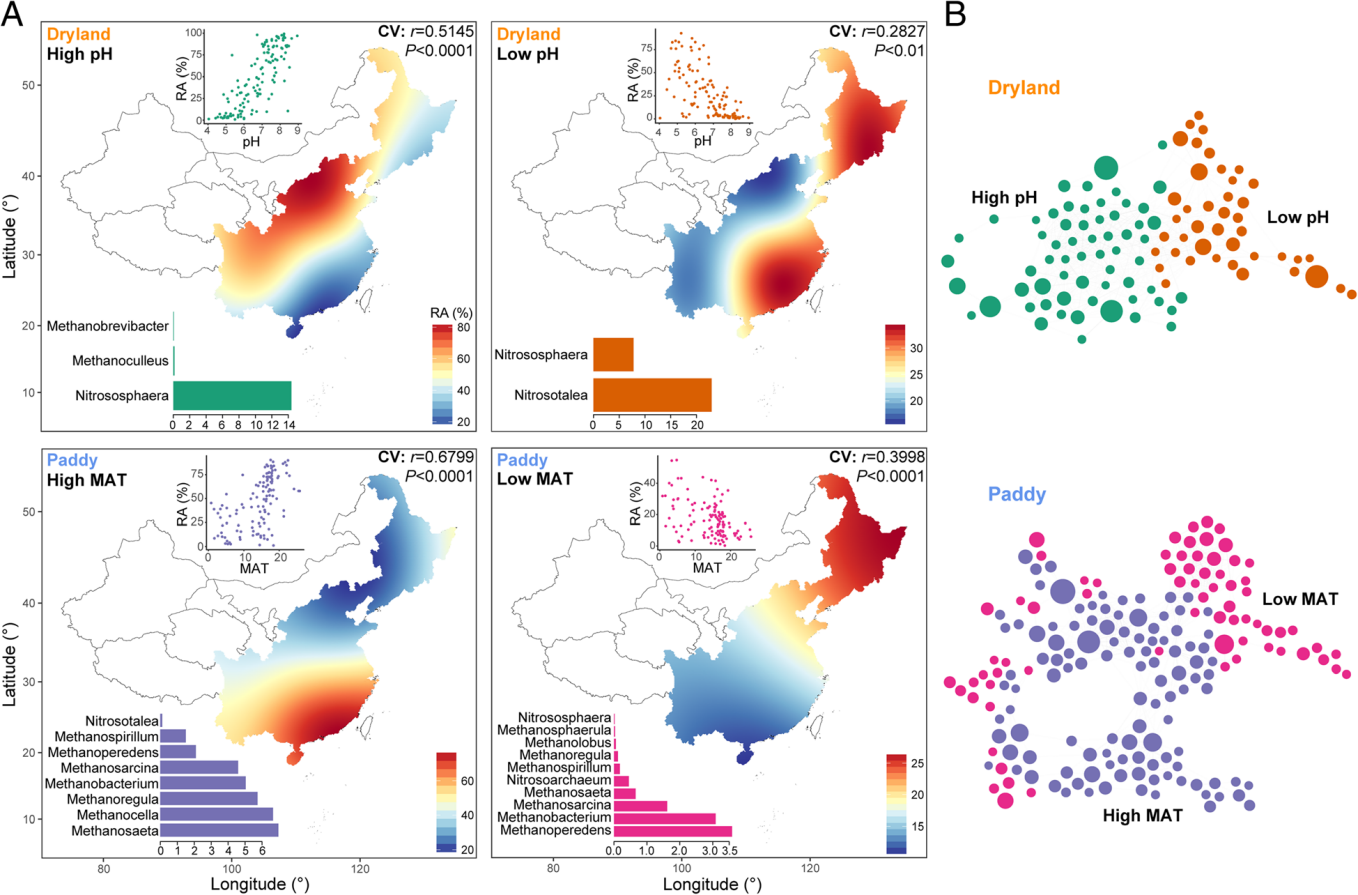
Continental atlases and co-occurrence patterns of dominant soil archaea with shared environmental preferences in agricultural fields across eastern China. Environmental preferences of dominant archaeal taxa were identified by Spearman correlations between the relative abundance of the taxa assigned to the two ecological clusters and their major environmental predictors, (1) high and (2) low pH for dryland cluster; (3) high and (4) low mean annual temperature (MAT) for paddy cluster (**a**). The atlas maps predicted the distributions of dominant archaeal taxa for each identified environmental preference across maize and rice fields, respectively. The cross-validation (“CV”) of the maps based on Pearson correlation between the predicted and observed values in each sampling site. Taxonomic compositions of dominant archaeal taxa at the genus level for the four sub-ecological clusters are displayed at the left-bottom of each graph. Network diagram with nodes (dominant archaeal taxa) colored by each sub-ecological cluster (**b**). The size of each node is proportional to the relative abundance of the taxa; the thickness of each connection between two nodes (edge) is proportional to the value of Spearman correlation coefficient

Furthermore, we explored the environmental preferences of the particular taxa within the abovementioned ecological clusters, namely sub-ecological clusters. Given that soil pH and MAT are the most important variables for respectively predicting archaeal assembly in maize and rice fields, we selected these two variables to identify the preferred sub-ecological attributes of dominant archaeal taxa corresponding to different ecological clusters. In total, the taxa were grouped into four sub-ecological clusters sharing environmental preferences, including (1) high pH and (2) low pH for dryland cluster, and (3) high MAT and (4) low MAT for paddy cluster (Additional file 1: Table S5). Our constructed spatial distribution maps provided estimates where we would expect the above clusters of dominant archaeal taxa to be most abundant (Fig. 4a). As expected, the low and high pH clusters were relatively abundant in known low and high pH regions, respectively, and similarly, the low or high MAT clusters were particularly abundant in areas known for their low or high MAT soils, respectively. In addition, strong relationships between environmental variables and relative abundances of corresponding ecological clusters indicated that these were reasonably well-defined ecological clusters (Fig. 4a). Going a step further, we estimated habitat preferences of the archaeal functional phylotypes. The ammonia-oxidizing *Nitrososphaera* and *Nitrosotalea* were favored in high and low pH environments, respectively. Most methanogens, however, were abundant in high MAT clusters. *Methanosaeta*, *Methanocella*, and *Methanoregula* were abundant in high MAT environments, while *Methanoperedens* was abundant in low MAT clusters. In addition, *Methanobacterium* and *Methanosarcina* had less specific habitat requirements, being present in both high and low MAT environments. Moreover, in the cross-validation—this used the abovementioned correlation network analysis for the co-occurrence of taxa—the nodes within the same ecological clusters (e.g., low MAT) were more connected; that is, dominant archaeal taxa sharing a particular habitat preference tended to co-occur with each other (Fig. 4b).

**Fig. 5:**
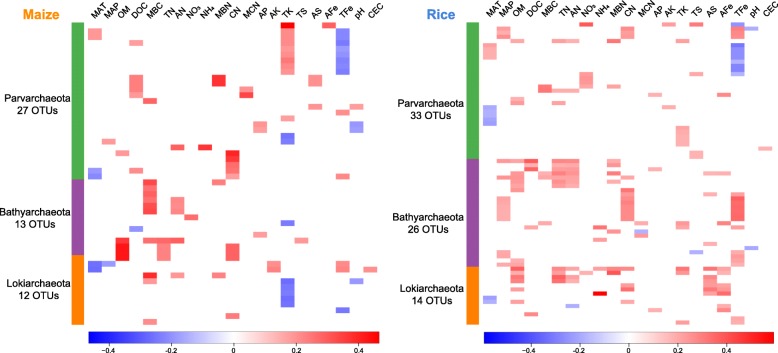
Spearman correlations between rare phyla and edaphic factors in maize and rice soils, displayed as heatmaps. Scale bars indicate correlation coefficients. Only significant correlations (*p* < 0.05) are shown. MAT, mean annual temperature; MAP, mean annual precipitation; OM, organic matter; DOC, dissolved organic carbon; MBC, microbial biomass carbon; TN, total nitrogen; AN, available nitrogen; NO_3_, nitrate-nitrogen; NH_4_, ammonium-nitrogen; MBN, microbial biomass nitrogen; CN, C:N ratio; MCN, microbial C:N ratio; AP, available phosphorus; AK, available potassium; TK, total potassium; TS, total sulfur; AS, available sulfur; AFe, available iron; TFe, total iron; and CEC, cation exchange capacity

We then correlated the dominant taxa to other soil nutrient properties and identified the major predictors for each dominant taxon (Additional file 1: Table S5 and Figure S9). Spearman correlation analysis revealed that there were contrasting environmental associations between methanogens and thaumarchaeotal genera (Additional file 1: Figure S9), indicating their distinct environmental preferences. In addition, NH_4_ and AS were strong positive predictors for the distributions of most methanogens whereas AFe had a strong negative effect on the relative abundance of OTUs assigned to *Nitrososphaera* (Additional file 1: Figure S9). Furthermore, several associations between phyla occurring at low abundance and environmental variables were significant (Spearman correlation, *p* < 0.05; Fig. 5). In particular, there were different association patterns between the maize and rice fields. For example, some taxa of Bathyarchaeota were positively correlated with TFe in rice fields, but their correlation was not significant in maize fields. Similarly, some lokiarchaeotal taxa were positively correlated with organic matter (OM), AS, and AFe in rice fields, yet not so in maize fields.

## Discussion

The widespread distribution of archaea in terrestrial ecosystems implies their potential participation and contribution to global biogeochemical cycles [23]. A previous study reported high variability in the general structure and relative abundance of dominant archaeal communities inhabiting soils at the global scale [5]. Agricultural soils are typically Anthrosols influenced by human activity, and their long-term tillage may alter the ecological diversity patterns of soil archaea and the ecosystem processes they sustain when compared with natural ecosystems. We fulfilled our study objectives, in that (1) environmental filtering plays a decisive role in driving the archaeal assembly in contrasting agricultural ecosystems; and (2) after identifying the dominant archaeal taxa and their habitat preferences, we built a continental atlas of soil archaeal communities that was distinct between dryland and wetland. These results could help us to predict the archaeal responses of agricultural ecosystems to anthropogenic disturbances and ongoing global environmental change.

Compared with dryland soils on which maize crops are cultivated, waterlogged paddy soils provide a rather unique habitat due to the oxygen-limited conditions generated there during frequent flooding events [24]. This management can sustain both aerobic and anaerobic taxa in alternating dry-wet paddy soils, leading to the greater archaeal *α*-diversity we found in rice than maize fields across eastern China. Several studies have shown that pH dominates the variation in archaeal diversity in forest and paddy soils [6, 12, 19]. However, we did not observe any significant relationships between soil pH and archaeal diversity, even though our soil samples spanned a wide range of pH values (4.03–9.89). We did find that archaeal diversity was negatively correlated with MAT in both maize and rice fields, yet positively correlated with the C:N ratio and AFe level in maize and rice fields, respectively. On the one hand, previous study has shown that microbial richness increased with temperature [25], according to the metabolic theory of ecology and latitudinal diversity gradient. On the other hand, a recent study investigated the global topsoil from natural terrestrial ecosystems, showing that bacterial diversity peaked at mid-latitudes and declined towards the poles and the equator, which is contrary to the typical latitudinal diversity gradient [26]. These contrary observations may be related to (1) the unique and more complex environment in human-managed agricultural fields compared with natural ecosystems and (2) the different microbial life-history between archaea (anaerobic) and bacteria (aerobic).

Earlier, we had observed marked differences in the edaphic properties between our maize and rice soil samples, even though most of them were collected from adjacent pairs of sites (in submission). This could help to explain why the assembly of archaeal communities differed considerably between maize and rice soils. We also found that *Nitrososphaera* and *Nitrosotalea* belonging to Thaumarchaeota, which are known as ammonia oxidizers [27, 28], were mainly dominant in maize soils. By contrast, we found that the Euryarchaeota, which contains many methanogens, predominate in rice soils; this result is consistent with the view that methanogenic Euryarchaeota are restricted to mostly anoxic environments [12, 29]. NH_4_ was an important contributor to the distributions of both Thaumarchaeota and Euryarchaeota. The negative correlations between Thaumarchaeota and NH_4_ could be due to high availability of NH_3_ to ammonia oxidizers in low-NH_4_ environments. By contrast, the positive correlations between Euryarchaeota and NH_4_ indicate that members of this phylum prefer environments with more available NH_4_-N. This resource dependence is probable given that methanogens source their nitrogen mainly from ammonium [24, 30]. Furthermore, sulfur also contributed to the distributions of these two phyla in rice soils; a plausible explanation for this result is that archaeal-driven ecosystem processes are involved in biological electron transferring in low-oxygen environments [14]. With regard to the phyla occurring at low abundances, bathyarchaeotal taxa were positively correlated with OM, TN, AN, and TFe in rice soils, indicating their potential participation in carbon, nitrogen, and iron cycling in anoxic environments [31]. Previous study has identified several syntenic genes with homology to those involved in iron oxidation in Parvarchaeota genomes and suggested their potential role in iron cycling [32]; this lent support to our observation that some parvarchaeotal taxa were correlated with TFe in rice and maize soils. The different environmental association patterns between the maize and rice fields indicate that despite low abundances, these archaeal species might have evolved diverse metabolic functions, enabling them to adapt to contrasting environmental conditions.

Distance-decay relationships describe decreasing community similarity with increasing geographic distance, and thus provide a directional model of variation in *β*-diversity across spatial scales [33, 34]. Importantly, the slopes of these relationships across habitats can differ, reflecting varying rates of species turnover in their habitats [35]. In our study, robust distance-decay relationships were established for archaeal communities in wetland and dryland agricultural ecosystems across a continental scale. The steeper distance-decay slope of rice fields suggests that their turnover of archaeal communities was faster than in rice fields. Since our pairwise adjacent sampling strategy covered similar spatial scales for both rice and maize fields, their different distance-decay slopes may be strongly correlated with environmental variability due to large spatial heterogeneity [36]. Rice soils can form unique, ephemeral habitats across local sites during long-term alternation of dry-wet conditions [24], thus generating more spatially structured archaeal communities across large scales. In addition, edaphic factors evidently affected archaeal community similarity in both maize and rice fields, suggesting the consequential roles of deterministic processes in driving archaeal community assembly. Geographic factors contributed a larger proportion of variation relative to edaphic factors in the archaeal *β*-diversity of rice soils than maize soils, indicating a stronger effect of stochastic processes in driving archaeal *β*-diversity of rice fields. This result is partly explained by the fact that frequent flooding management generates more similar communities in sites nearer to each other than in those further apart; this should enhance inherent stochastic processes operating in rice fields, such as drift and dispersal limitation.

A global inventory of dominant soil bacterial phylotypes consisted of a small subset of phylotypes which accounted for almost half of the 16S rRNA sequences recovered from soils [22]. In the present study, the dominant archaeal taxa (amounting ~ 10% of total taxa) identified in maize and rice fields accounted for more than 90% of the archaeal sequences. We also mapped the spatial distributions of these dominant archaeal taxa in the soil samples. These results suggest that, much like soil bacteria in natural terrestrial ecosystems [22], there are predictable environmental gradients and pockets for dominant archaeal taxa in agricultural soils at the continental scale, so that their spatial distributions can be predicted reliably by a continental atlas. Distinct spatial distributions were generated for the two ecological clusters of dominant archaeal taxa, separated based on their preferences of dry or waterlogged environments. The dryland cluster dominated by thaumarchaeotal taxa had a greater relative abundance in northern than southern counterparts, whereas the dominant taxa that preferred waterlogged environments, mainly euryarchaeotal OTUs, were more abundant in southern regions. Rice fields are man-made methanogenic environments, harboring a variety of euryarchaeotal methanogens [37]. This distinct continental atlas implies the contrasting habitat preferences and ecological assembly of dominant soil archaeal taxa in wetland and dryland agricultural ecosystems. In determining their habitat preferences, our results suggest that the dominant archaeal taxa tended to co-occur with others sharing the same habitat requirements. These results imply that strong phylogenetic linkages, manifested as a cluster of robust co-occurrence correlations, reflect complex associations with the same ecological attributes.

Habitat preferences of dominant archaeal taxa are associated with their ecological characteristics, such as physiological capabilities [38, 39]. By focusing on the archaeal functional phylotypes—e.g., ammonia oxidizers and methanogen—we were able to predict the environmental conditions that favored them. For example, our finding that *Nitrososphaera* and *Nitrosotalea* preferred high-pH and low-pH environments, respectively, is supported by other works showing that these two genera are correspondingly alkaliphilic [27] and acidophilic [28]. These consistent lines of evidence from other investigations strengthen the overall reliability of our predicted environmental preferences for these dominant archaeal taxa. We also observed that most methanogens preferred to inhabit high temperature regions. Temperature was proven to be an important factor affecting the structure and function of soil methanogenic communities [40]. Our field study provides information to predict the preferred environmental conditions (e.g., low or high pH) of desired archaeal taxa and to enrich particular dominant taxa in vitro, thereby increasing our ability to successfully cultivate them.

## Conclusions

This empirical study provides a detailed and systematic survey of soil archaeal communities in maize and rice fields across eastern China. The results indicate that environmental filtering plays a decisive role in driving the distinct continental atlases of soil archaeal communities’ wetland and dryland agricultural ecosystems. Thaumarchaeota and Euryarchaeota phyla dominated the maize and rice cultivated fields, respectively, indicating the existence of distinct archaeal-driven ecosystem processes within these two agricultural ecosystems. By identifying the dominant archaeal taxa along with their habitat preferences, we built a robust continental atlas of soil archaeal spatial distribution and ecological diversity, thus improving our ability to predict the responses of agricultural ecosystems to anthropogenic disturbances. Overall, our study implies that instead of considering tens of thousands of members, future research ought to narrow its focus upon the few hundred dominant taxa in soil microbial communities. This approach is also critical for quickly and accurately forecasting the ecological consequences of ongoing global environmental change.

## Methods

### Soil sampling

A total of 132 locations were selected from agricultural fields under long-term cultivation with maize and rice across eastern China. These included 117 paired sites, 8 maize-only, and 7 rice-only sites, amounting to 125 maize and 124 rice soil samples (Additional file 1: Figure S1). Each paired site consisted of a maize field adjacent to a rice field (less than 5 km apart). The soils at all the sites were sampled during the planting season (July–September 2017). Three plots (each 100 m^2^) at each site were randomly selected, from which five soil cores per plot were taken at a depth of 0–15 cm and combined. These plot-level samples were sieved through a 2.0-mm mesh to remove plant debris and rocks and then mixed thoroughly for the three plots on a per site basis to generate the final composite soil samples.

Standard soil testing procedures were followed to measure soil pH, CEC, and nutrient factors—namely, OM, dissolved organic carbon (DOC), total nitrogen (TN), available nitrogen (AN), nitrate-nitrogen (NO_3_), ammonium-nitrogen (NH_4_), total phosphorus (TP), available phosphorus (AP), total potassium (TK), available potassium (AK), microbial biomass carbon (MBC), and microbial biomass nitrogen (MBN)—as well as a few factors involved in biological electron transfers, such as TFe, AFe, TS, and AS, according to the literature [15, 41]. Climatic variables, including MAT and mean annual precipitation (MAP), for each sampling site were obtained using its coordinates from the WorldClim database (www.worldclim.org).

### Illumina sequencing of the 16S rRNA gene

Total genomic DNA was extracted from each soil sample using the MP FastDNA SPIN Kit [for soil] (MP Biomedicals, Solon) as per the manufacturer’s instructions. The archaeal 16S rRNA gene was PCR-amplified using the primers Arch519F (CAGCCGCCGCGGTAA) / Arch915R (GTGCTCCCCCGCCAATTCCT) that were combined with adapter sequences and barcode sequences. Purified amplicons were sequenced on a HiSeq2500 platform (Illumina Inc., San Diego, USA). Paired-end reads were first merged using FLASH software and then quality filtered according to the procedure described by Caporaso et al. [42]. Chimera detection and removal was accomplished using the USEARCH tool in the UCHIME algorithm [43]. Sequences were split into groups according to taxonomy and assigned to OTUs at a 3% dissimilarity level (i.e., 97% similarity) using the UPARSE pipeline [43]. Those OTUs lacking more than two sequences were removed; representative sequences of the remaining OTUs were classified using the SILVA database release 128.

### Statistical analyses

All statistical analyses were conducted in the R environment (v3.5.1; http://www.r-project.org/) using “vegan” [44], “igraph” [45], “Hmisc” [46], “automap” [47] “ggplot2” [48], and “gplots” [49] packages, unless otherwise indicated.

After removing the bacterial and unknown OTUs, the samples were subsampled to a minimum number of sequences (36,880) to control the sampling effort. The *α*-diversity (expressed as OTU richness and Shannon index) of each sample was calculated, and the *β*-diversity was estimated (based on Bray-Curtis distances between samples). The geographical distances among the sampling sites were calculated from the sampling coordinates. To visualize the relationships of archaeal communities from maize and rice soils, NMDS analysis was performed based on Bray-Curtis distances by using the “metaMDS” function of the “vegan” package [44]. To determine significant differences in archaeal *β*-diversity between the maize and rice fields, ANOSIM and ADONIS were carried out using the “anosim” and “adonis” function of the “vegan” package [44].

Distance-decay relationships were calculated as the linear least-squares regression relationships between geographic distance and community similarity (based on 1 – [dissimilarity of the Bray-Curtis distance metric]). To disentangle the relative importance of edaphic, geographic, and climatic variables for archaeal community assembly, a variation-partitioning analysis was performed using “varpart” function of the “vegan” package [44]. To limit co-linearity effects between variables, variable clustering was used to assess the redundancy of environmental variables. The analysis was performed and plotted using “varclus” in “Hmisc” R package [46]. Geographic variables were derived from spatial coordinates by using the principal coordinates of neighbor matrices (PCNM) procedure to capture all the detectable spatial scales in the dataset [50], conducted by “pcnm” function of the “vegan” package [44]. Then, a distance-based linear model and forward selection procedure based on the Bray-Curtis distance matrix was used to select the respective subsets of edaphic, climatic, and geographic variables [51, 52]. The forward selection was stopped if an insignificant alpha level (*p* value > 0.05) was reached, or if there was no model improvement seen in the variation being explained (*R*^2^) after adding any additional variables. The statistical significance of each group of explanatory variables via partitioning was evaluated with a permutation test. Variation partitioning was performed with adjusted *R*^2^ values to determine the proportion of variation in the archaeal communities explained by the fitted model. Additionally, the results for the selected edaphic variables were displayed by a constrained analysis of principal coordinates based on Bray-Curtis distances using the “capscale” function of the “vegan” package [44].

The most common and ubiquitous archaeal taxa across the agricultural fields were identified using the criteria of Delgado-Baquerizo et al. [22] with slight modifications: (1) selecting only highly abundant OTUs, i.e., those with relative abundance that ranked in the top 20% across all samples; and (2) keeping those OTUs occurring in more than half (> 50%) of all the 249 soil samples. Hence, with these two criteria, the OTUs that were abundant and widely present across soil samples were considered. Given the distinct archaeal assembly patterns between maize and rice fields, we determined the ecological preferences of dominant archaeal taxa by focusing on whether they preferred a waterlogged environment. Spearman correlations identified the groups of dominant taxa with shared habitat preferences. The dominant archaeal taxa were grouped into two ecological clusters sharing habitat preferences for (1) dryland and (2) paddy. Furthermore, since soil pH and MAT are the most important variables for respectively predicting archaeal assembly in maize and rice fields, we selected on these two variables to identify the preferred sub-ecological attributes of the dominant archaeal taxa corresponding to different ecological clusters. In total, the taxa were grouped into four sub-ecological clusters sharing environmental preferences via Spearman correlations, including (1) high pH and (2) low pH for dryland cluster, and (3) high MAT and (4) low MAT for paddy cluster. To build predictive maps of the spatial distributions of the core bacterial taxa, we used a kriging interpolation method to estimate the relative abundance of each ecological cluster in maize and rice fields, respectively. This analysis was performed in the “automap” package [47], which automates the interpolation process by automatically estimating a semivariogram and performing kriging interpolation. We cross-validated our maps using “autoKrige.cv” in “automap” package [47]. The predicted relative abundances of each cluster were extracted for the selected soil samples and then correlated to the observed values in the corresponding sites based on Pearson correlation analysis. The Pearson correlation coefficient and *p* value were shown in the map. Co-occurrence networks were constructed to evaluate whether dominant archaeal taxa within a particular ecological cluster co-occurred more often. To do this, robust Spearman correlations between any two dominant OTUs were used (defined as those with rho coefficients > 0.6 and FDR-corrected *p* values < 0.01). This formed a correlation network, in which each node represents one OTU, and each edge represents a strong and significant correlation between two nodes. Networks were visualized using the interactive Gephi platform [53].

## Additional file

Additional file 1: **Figure S1.** Geographical location of the sampling sites for agricultural soils across eastern China, including 117 paired, 8 maize-only, and 7 rice-only sites. **Figure S2.** Variation in the archaeal *α*-diversity indices [operational taxonomic unit (OTU) richness and Shannon index] between maize and rice soils. **Figure S3.** Relationships between archaeal Shannon index and environmental variables in each pair of maize (**A** and **B**) and rice (**C** and **D**) soils, estimated by linear least-squares regression. **Figure S4**. Variation in the relative abundance of archaeal phyla between maize and rice soils. **Figure S5.** Predicted spatial distributions of Euryachaeota and Thaumarchaeota in maize and rice soils. **Figure S6.** Variation in the relative abundance of archaeal orders between maize and rice soils. **Figure S7.** Variation in the relative abundance of archaeal genera between maize and rice soils. **Figure S8.** Cluster analysis of the measured environmental variables in maize and rice fields. **Figure S9.** Environmental contributions to the distributions of dominant archaeal taxa in maize and rice soils. **Table S1.** Variation explained by environmental variables in the regression models for archaeal Shannon index in maize and rice fields across eastern China. **Table S2.** Variation explained by environmental variables in the regression models for the relative abundance of Euryarchaeota and Thaumarchaeota in maize and rice fields. **Table S4.** ANOVA of environmental factors correlated with archaeal *β*-diversity in rice soil. **Table S5.** List of soil dominant archaeal taxa in agricultural fields across eastern China. (ZIP 5765 kb)
