# Supplementary material for: Environmental filtering drives distinct continental atlases of soil archaea between dryland and wetland agricultural ecosystems
Source: Microbiome. 2019 Feb 1;7:15. doi: 10.1186/s40168-019-0630-9 (PMC6359761; doi:10.1186/s40168-019-0630-9)
Supplement: Supplementary file 1 — Figure S1. Geographical location of the sampling sites for agricultural soils across eastern China, including 117 paired, 8 maize-only, and 7 rice-only sites. Figure S2. Variation in the archaeal α-diversity indices [operational taxonomic unit (OTU) richness and Shannon index] between maize and rice soils. Figure S3. Relationships between archaeal Shannon index and environmental variables in each pair of maize (A and B) and rice (C and D) soils, estimated by linear least-squares regression. Figure S4. Variation in the relative abundance of archaeal phyla between maize and rice soils. Figure S5. Predicted spatial distributions of Euryachaeota and Thaumarchaeota in maize and rice soils. Figure S6. Variation in the relative abundance of archaeal orders between maize and rice soils. Figure S7. Variation in the relative abundance of archaeal genera between maize and rice soils. Figure S8. Cluster analysis of the measured environmental variables in maize and rice fields. Figure S9. Environmental contributions to the distributions of dominant archaeal taxa in maize and rice soils. Table S1. Variation explained by environmental variables in the regression models for archaeal Shannon index in maize and rice fields across eastern China. Table S2. Variation explained by environmental variables in the regression models for the relative abundance of Euryarchaeota and Thaumarchaeota in maize and rice fields. Table S4. ANOVA of environmental factors correlated with archaeal β-diversity in rice soil. Table S5. List of soil dominant archaeal taxa in agricultural fields across eastern China. (ZIP 5765 kb) [file 40168_2019_630_MOESM1_ESM.zip › Additional file1.docx]

**Supplementary material for**

**Environmental filtering drives distinct continental atlases of soil archaea between dryland and wetland agricultural ecosystems**

**Running title**: Continental atlases of agro-soil archaea

Shuo Jiao^1^, Yiqin Xu^1^, Jie Zhang^1^ and Yahai Lu^1,^*

^1^College of Urban and Environmental Sciences, Peking University, Beijing 100871, P. R. China

*Correspondence and requests for materials should be addressed to Y.L.

Tel: +86 10 6275 5683; Fax: +86 10 6275 5683

E-mail: luyh@pku.edu.cn

**Fig. S1** Geographical location of the sampling sites for agricultural soils across eastern China, including 117 paired, 8 maize-only, and 7 rice-only sites


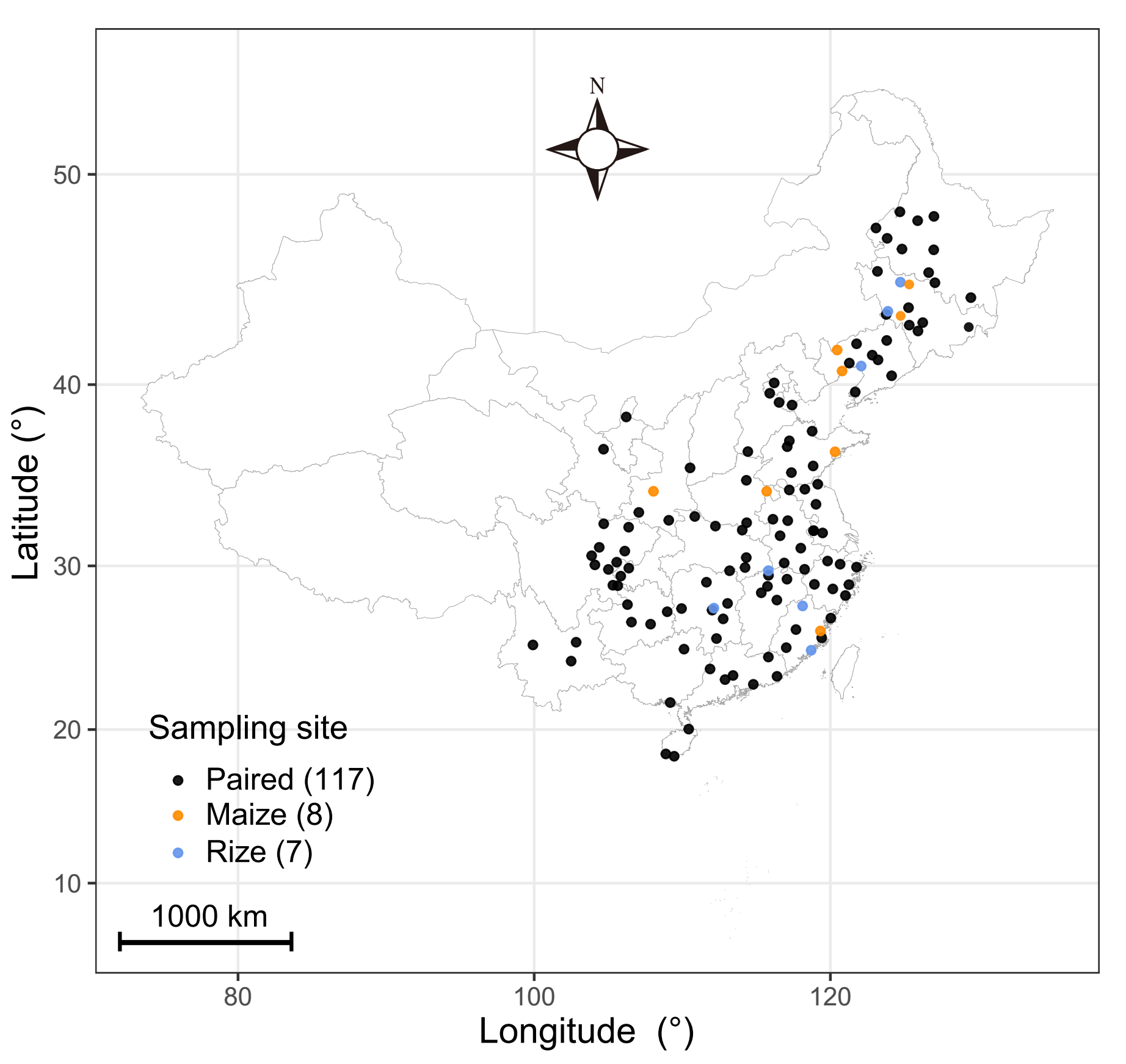


**Fig. S2** Variation in the archaeal *α*-diversity indices [operational taxonomic unit (OTU) richness and Shannon index] between maize and rice soils. Asterisks indicate significant difference (***, *p* < 0.001; Wilcoxon rank-sum test).


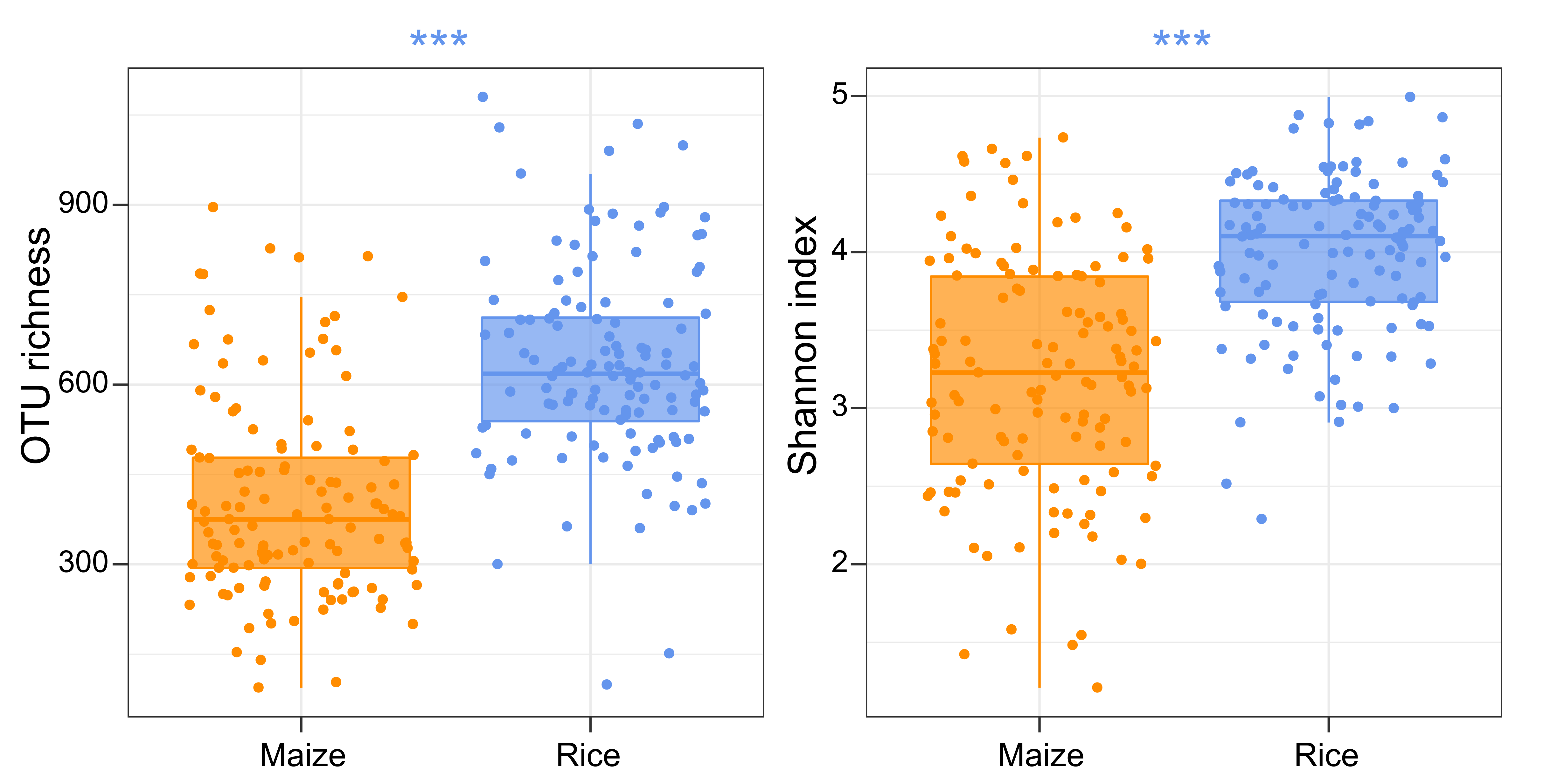


**Fig. S3** Relationships between archaeal Shannon index and environmental variables in each pair of maize (**A** and **B**) and rice (**C** and **D**) soils, estimated by linear least-squares regression


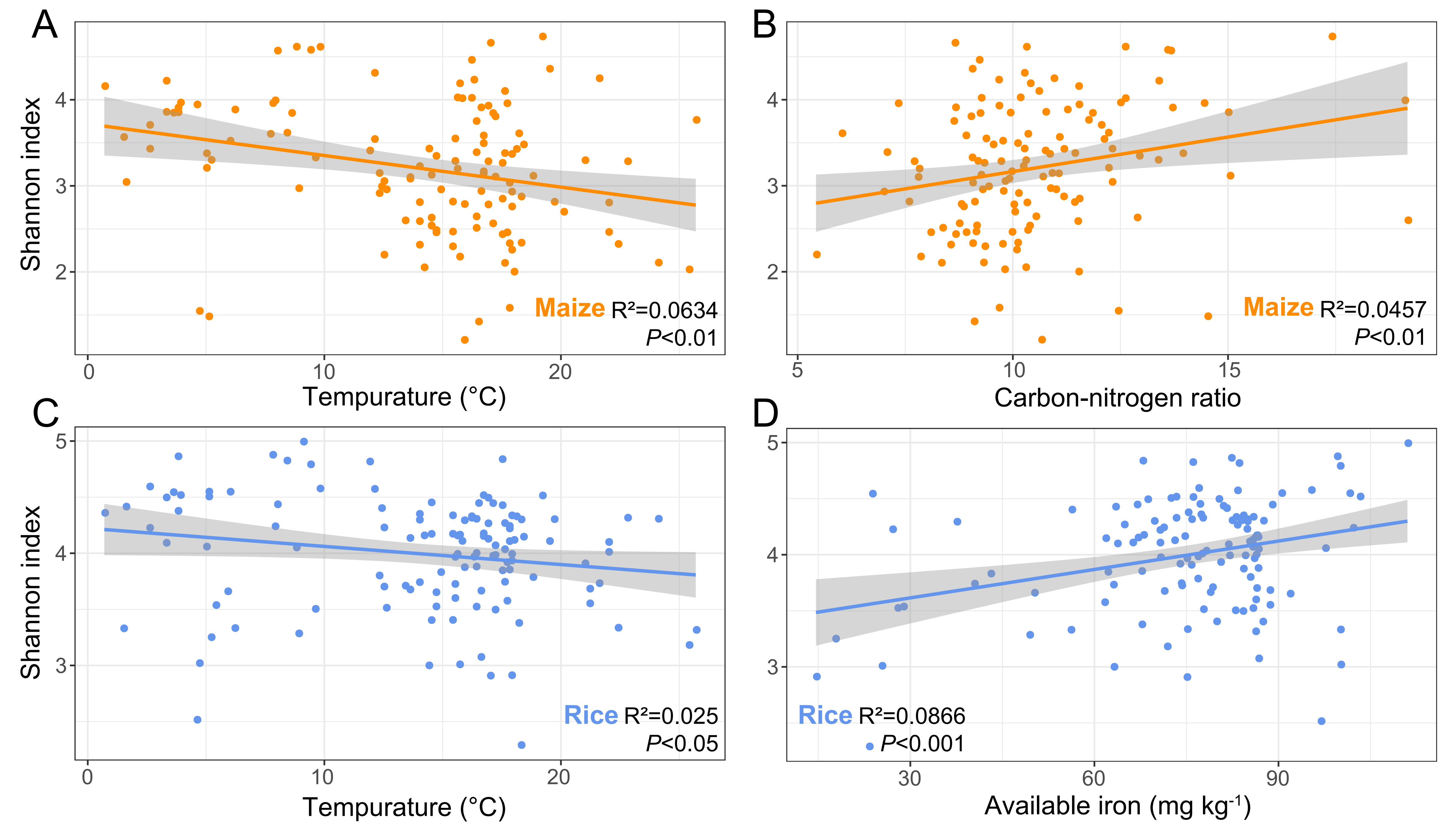


**Fig. S4** Variation in the relative abundance of archaeal phyla between maize and rice soils. Asterisks indicate significant difference (***, *p* < 0.001; Wilcoxon rank-sum test)


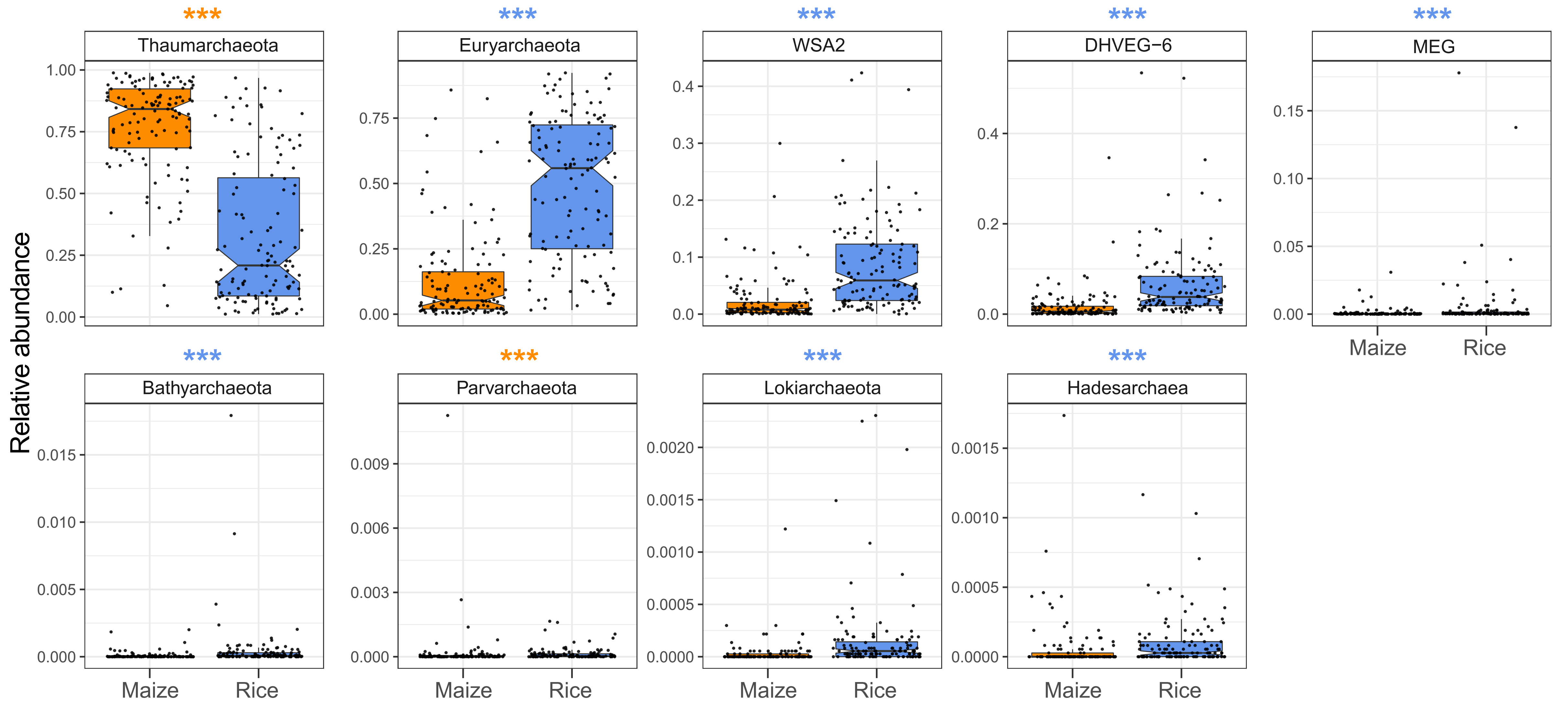


**Fig. S5** Predicted spatial distributions of Euryachaeota and Thaumarchaeota in maize and rice soils. The cross-validation (“CV”) of the maps based on Pearson correlation between the predicted and observed values in each sampling site.


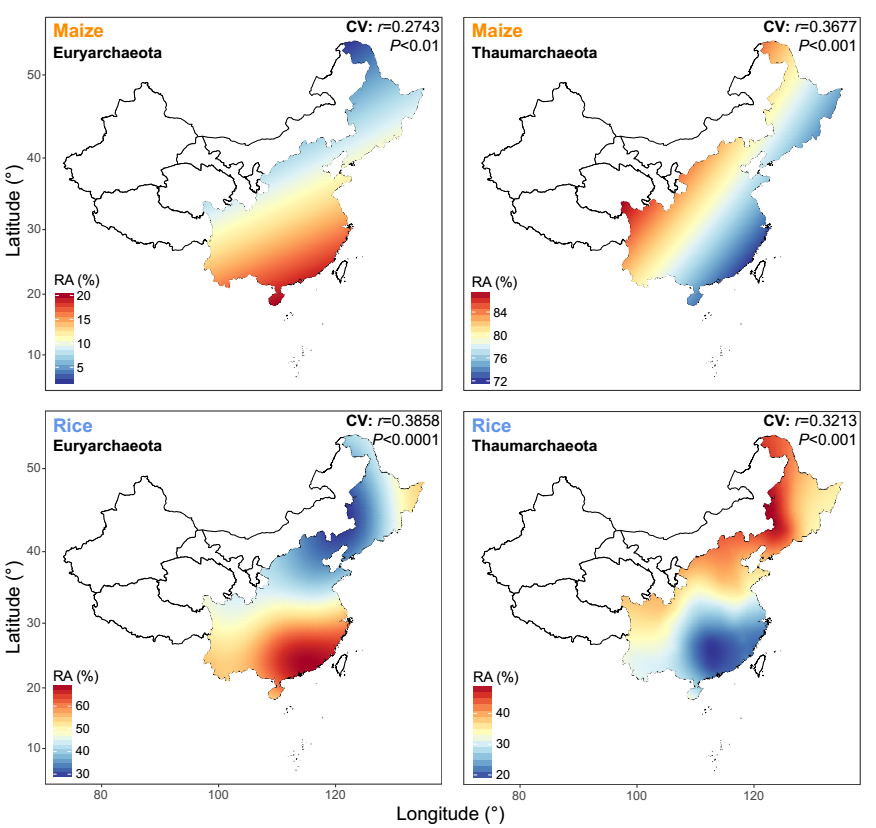


**Fig. S6** Variation in the relative abundance of archaeal orders between maize and rice soils. Asterisks indicate significant difference (***, *p* < 0.001; Wilcoxon rank-sum test).


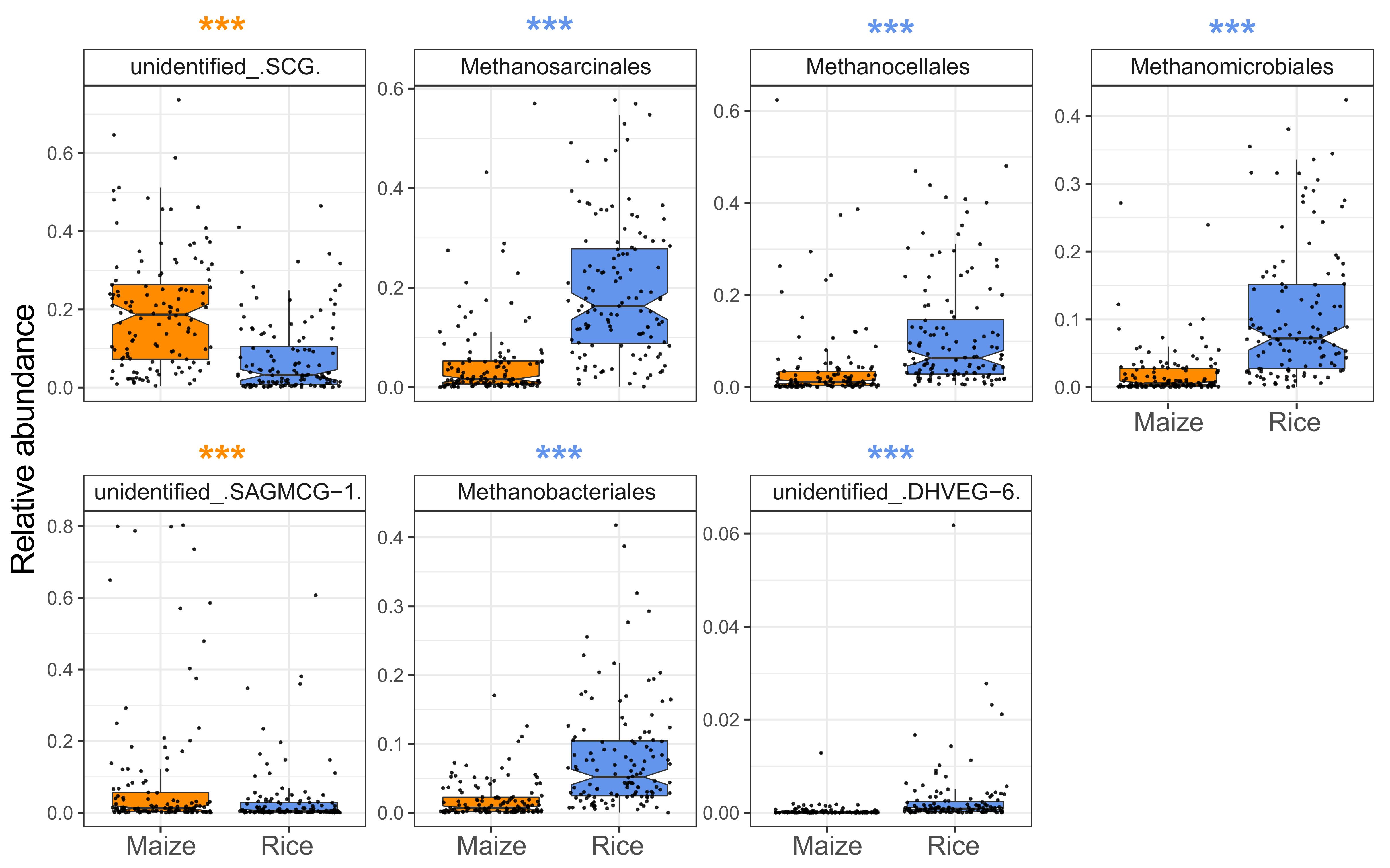


**Fig. S7** Variation in the relative abundance of archaeal genera between maize and rice soils. Asterisks indicate significant difference (***, *p* < 0.001; Wilcoxon rank-sum test).


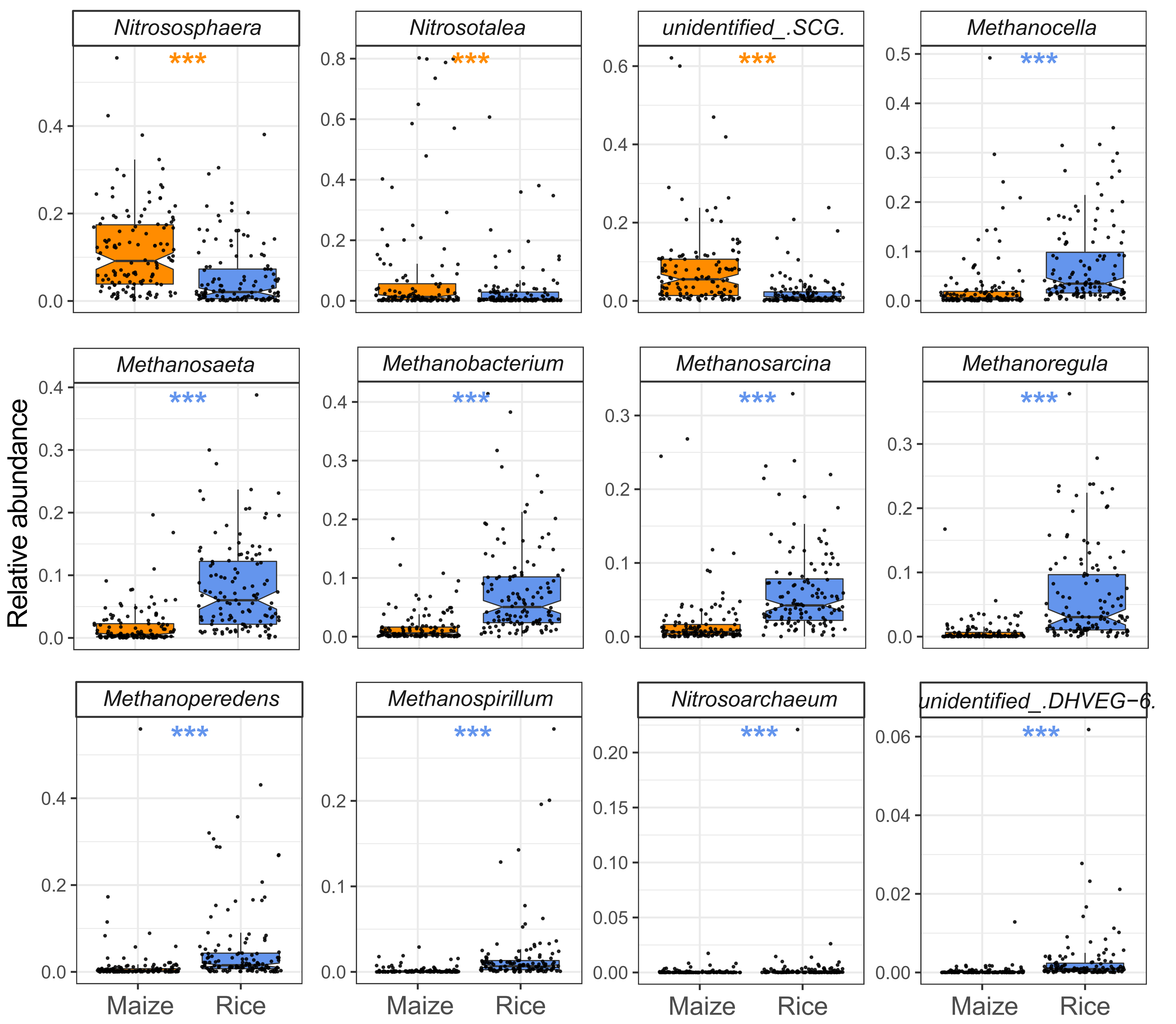


**Fig. S8** Cluster analysis of the measured environmental variables in maize and rice fields. The analysis was performed and plotted using “varclus” in “Hmisc” R package.


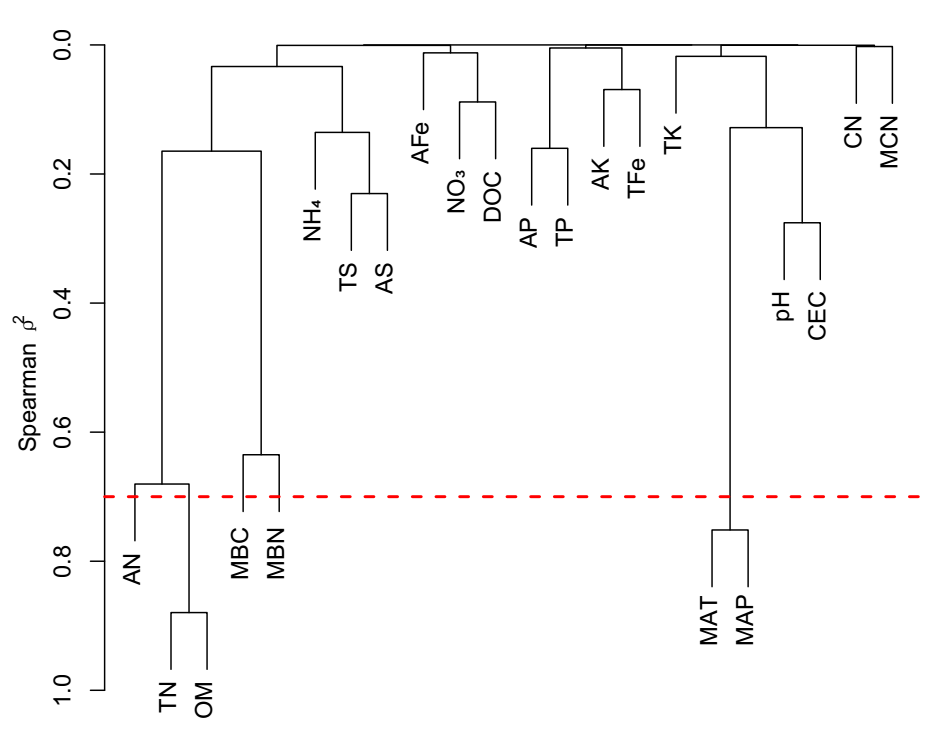


**Fig. S9** Environmental contributions to the distributions of dominant archaeal taxa in maize and rice soils. Correlation and multiple regression model for the major taxonomic categories of the top 40 most dominant archaeal taxa that could be assigned to the genus level were shown in heatmap. Circle size represents the variable importance (i.e., the proportion of explained variance calculated via multiple regression modeling and variance decomposition analysis). Colors represent Spearman correlations. AK, available potassium; TN, total nitrogen; AP, available phosphorus; AN, available nitrogen; NO_3_, nitrate-nitrogen; NH_4_, ammonium-nitrogen; TP, total phosphorus; TK, total potassium; OM, organic matter; CEC, cation exchange capacity; DOC, dissolved organic carbon; MBC, microbial biomass carbon; MBN, microbial biomass nitrogen; TS, total sulfur; AS, available sulfur; AFe, available iron; TFe, total iron; CN, carbon/nitrogen ratio; and MCN, microbial carbon/nitrogen ratio.


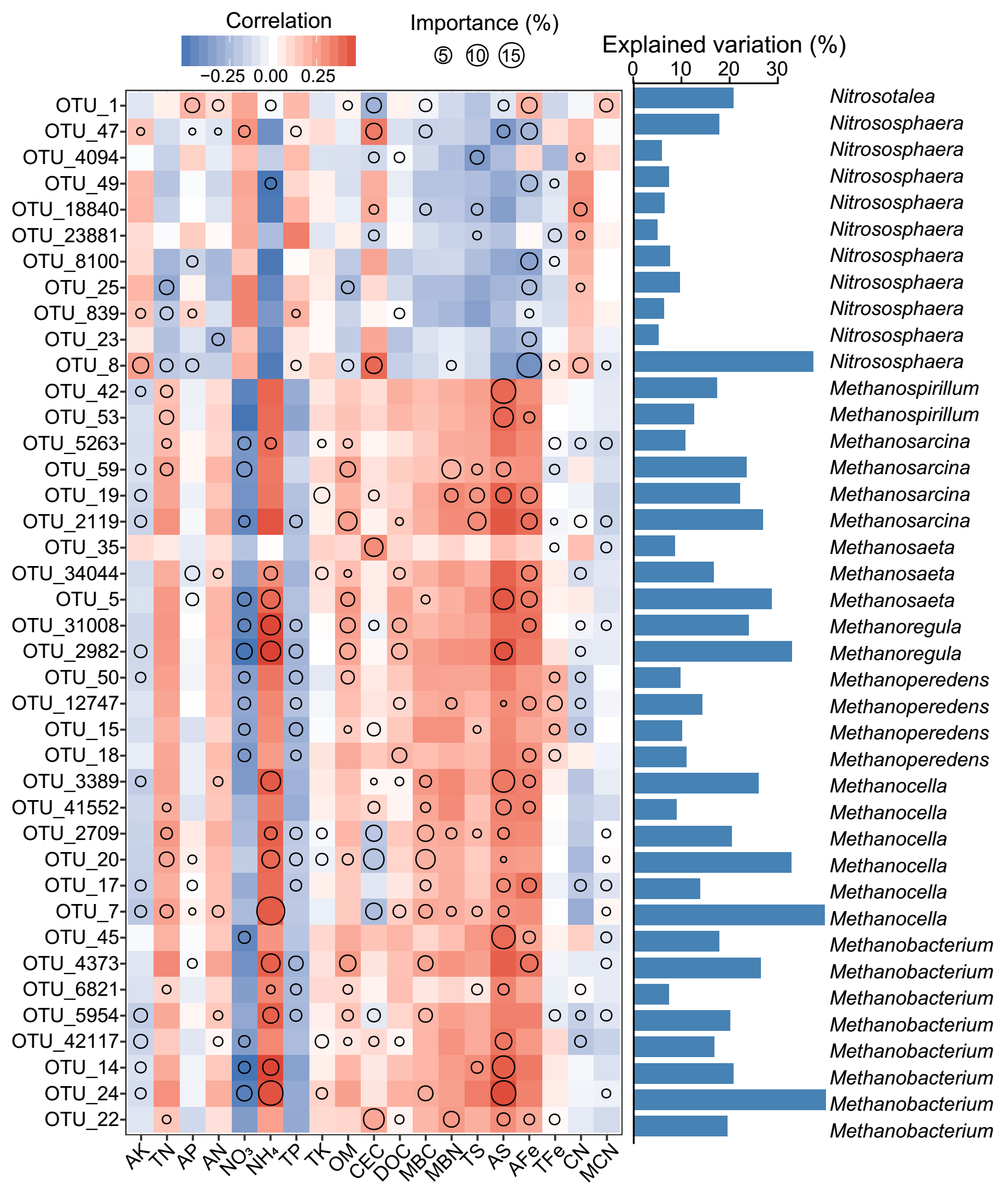


**Table S1** Variation explained by environmental variables in the regression models for archaeal Shannon index in maize and rice fields across eastern China. MAT, mean annual temperature; MAP, mean annual temperature precipitation; AFe, available iron; TN, total nitrogen; NH_4_, ammonium-nitrogen; MBC, microbial biomass carbon; MBN, microbial biomass nitrogen; TS, total sulfur; and C/N, carbon/nitrogen ratio

| **Environmental factor** | **Maize** | **Rice** |
| --- | --- | --- |
| MAT | 4.98% | 5.05% |
| MAP |  | 1.41% |
| AFe | 2.12% | 8.70% |
| TN | 2.26% |  |
| NH_4_ | 0.83% |  |
| pH | 1.42% |  |
| MBC | 1.69% | 3.32% |
| MBN | 0.61% |  |
| TS | 0.50% |  |
| C/N | 4.26% |  |
| **Total** | **18.66%** | **18.47%** |

NA, not statistically significant (*p* > 0.05)

**Table S2** Variation explained by environmental variables in the regression models for the relative abundance of Euryarchaeota and Thaumarchaeota in maize and rice fields. NH_4_, ammonium-nitrogen; TS, total sulfur; AS, available sulfur; OM, organic matter; MAT, mean annual temperature; TP, total phosphorus; AFe, available iron; CEC, cation exchange capacity; C/N, carbon/nitrogen ratio; NO_3_, Nitrate-nitrogen; AK, available potassium; and DOC, dissolved organic carbon

| **Environmental variable** | **Maize** | | **Rice** | |
| --- | --- | --- | --- | --- |
|  | **Euryarchaeota** | **Thaumarchaeota** | **Euryarchaeota** | **Thaumarchaeota** |
| NH_4_ | 15.72% | 10.50% | 10.12% |  |
| TS | 2.16% | 2.07% |  | 9.54% |
| AS | 2.28% | 1.63% | 8.46% | 8.55% |
| OM | 4.26% | 5.87% | 4.84% |  |
| MAT | 3.03% | 0.95% |  |  |
| TP | 3.49% | 3.28% |  |  |
| AFe | 1.97% |  | 2.92% | 4.73% |
| pH | 1.63% |  |  | 6.90% |
| CEC | 0.96% |  | 5.24% |  |
| C/N |  |  | 4.32% | 2.06% |
| NO_3_ |  |  | 5.37% | 5.24% |
| AK |  |  | 3.14% |  |
| DOC |  |  |  | 3.29% |
| **Total** | **35.52%** | **24.31%** | **44.42%** | **40.32%** |

NA, not statistically significant (*p* > 0.05)

**Table S3** ANOVA of environmental factors correlated with archaeal *β*-diversity in maize soils. MAT, mean annual temperature; CEC, cation exchange capacity; NH_4_, ammonium-nitrogen; and AFe, available iron

| **Factor** | **df** | **Variance** | **Pseudo-F** | **p-value** |
| --- | --- | --- | --- | --- |
| pH | 1 | 5.35 | 15.03 | 0.001 |
| MAT | 1 | 2.16 | 6.08 | 0.001 |
| CEC | 1 | 0.67 | 1.88 | 0.023 |
| NH_4_ | 1 | 0.66 | 1.85 | 0.014 |
| AFe | 1 | 0.67 | 1.87 | 0.007 |
| Residual | 119 | 42.33 |  |  |
|  |  |  |  |  |
| **Axis** | **df** | **Variance** | **Pseudo-F** | **p-value** |
| CAP1 | 1 | 5.46 | 15.35 | 0.001 |
| CAP2 | 1 | 2.26 | 6.35 | 0.001 |
| CAP3 | 1 | 0.71 | 2.00 | 0.003 |
| CAP4 | 1 | 0.58 | 1.62 | 0.012 |
| CAP5 | 1 | 0.50 | 1.40 | 0.047 |
| Residual | 119 | 42.33 |  |  |

df = degree of freedom

Permutations = 999

Overall model significance: *Pseudo-F* = 5.344, *p* = 0.001

**Table S4** ANOVA of environmental factors correlated with archaeal *β*-diversity in rice soil. MAT, mean annual temperature; AS, available sulfur; NO_3_, Nitrate-nitrogen; TFe, total iron; TN, total nitrogen; and AFe, available iron

| **Factor** | **df** | **Variance** | **Pseudo-F** | **p-value** |
| --- | --- | --- | --- | --- |
| MAT | 1 | 2.83 | 9.45 | 0.001 |
| pH | 1 | 2.08 | 6.95 | 0.001 |
| AS | 1 | 1.35 | 4.53 | 0.001 |
| NO_3_ | 1 | 0.74 | 2.47 | 0.001 |
| TFe | 1 | 0.65 | 2.18 | 0.002 |
| TN | 1 | 0.60 | 1.99 | 0.004 |
| AFe | 1 | 0.53 | 1.78 | 0.004 |
| Residual | 116 | 34.70 |  |  |
|  |  |  |  |  |
| **Axis** | **df** | **Variance** | **Pseudo-F** | **p-value** |
| CAP1 | 1 | 3.98 | 13.31 | 0.001 |
| CAP2 | 1 | 1.67 | 5.58 | 0.001 |
| CAP3 | 1 | 1.28 | 4.28 | 0.001 |
| CAP4 | 1 | 0.66 | 2.20 | 0.001 |
| CAP5 | 1 | 0.50 | 1.67 | 0.012 |
| CAP6 | 1 | 0.38 | 1.29 | 0.094 |
| CAP7 | 1 | 0.31 | 1.02 | 0.356 |
| Residual | 116 | 34.70 | NA | NA |

df = degree of freedom

Permutations = 999

Overall model significance: *Pseudo-F* = 4.0688, *p* = 0.001

**Table S5** List of soil dominant archaeal taxa in agricultural fields across eastern China. This list contains information on the relative abundance, ubiquity, ecological cluster, sub-ecological cluster, taxonomic identity and the variation explained by environmental predictors in the regression models for each dominant archaeal taxa.

***Table S5*** *are available online as separate .XLS files under the Supporting Materials for this article*
